# Age-Related Differences in Pro-active Driving Behavior Revealed by EEG Measures

**DOI:** 10.3389/fnhum.2018.00321

**Published:** 2018-08-07

**Authors:** Stephan Getzmann, Stefan Arnau, Melanie Karthaus, Julian Elias Reiser, Edmund Wascher

**Affiliations:** Leibniz Research Centre for Working Environment and Human Factors, Technical University of Dortmund, Dortmund, Germany

**Keywords:** EEG, aging, proactive driving, mental effort, workload, alpha oscillations, theta oscillations, event-related potentials

## Abstract

Healthy aging is associated with a decline in cognitive functions. This may become an issue when complex tasks have to be performed like driving a car in a demanding traffic situation. On the other hand, older people are able to compensate for age-related deficits, e.g., by deploying extra mental effort and other compensatory strategies. The present study investigated the interplay of age, task workload, and mental effort using EEG measures and a proactive driving task, in which 16 younger and 16 older participants had to keep a virtual car on track on a curvy road. Total oscillatory power and relative power in Theta and Alpha bands were analyzed, as well as event-related potentials (ERPs) to task-irrelevant regular and irregular sound stimuli. Steering variability and Theta power increased with increasing task load (i.e., with shaper bends of the road), while Alpha power decreased. This pattern of workload and mental effort was found in both age groups. However, only in the older group a relationship between steering variability and Theta power occurred: better steering performance was associated with higher Theta power, reflecting higher mental effort. Higher Theta power while driving was also associated with a stronger increase in reported subjective fatigue in the older group. In the younger group, lower steering variability came along with lower ERP responses to deviant sound stimuli, reflecting reduced processing of task-irrelevant environmental stimuli. In sum, better performance in proactive driving (i.e., more alert steering behavior) was associated with increased mental effort in the older group, and higher attentional focus on the task in the younger group, indicating age-specific strategies in the way younger and older drivers manage demanding (driving) tasks.

## Introduction

Healthy aging is usually associated with a decline in sensory, cognitive and motor functions (Park, 2000; Lindenberger and Ghisletta, 2009). All these abilities are required when complex tasks have to be performed, like driving a car through dense city traffic or on a monotonous road where attention towards driving-related events has to be kept over a longer period of time. In fact, car driving can be regarded as a prototypical example of a complex task in which an adequate interplay of information intake, cognitive processing, and motor responses is necessary. Each of these instances may be prone to age-related deficits. For example, the sensory intake may be reduced due to vision problems or responses to unexpected critical traffic events may be slowed down due to motor impairments (Anstey and Wood, 2011; Park et al., 2011). In addition, driving under challenging conditions may lead to a greater mental workload for older drivers (Cantin et al., 2009). Besides these negative consequences of aging, increasing driving experience and concurrent emergence of enhanced driving strategies are advantages that can help older drivers to manage complex traffic situations. Furthermore, it is known that older drivers are able to compensate for age-related decline, at least in part. On the one hand, compensation comprises strategies to adapt driving behavior to individual abilities, for example, by reducing driving speed and avoiding driving in the rain or for long periods of time (e.g., Molnar and Eby, 2008; Meng and Siren, 2012). On the other hand, older adults tend to increase their mental effort in order to counteract age-related neurocognitive decline (Cabeza et al., 2002). In the driving context, older drivers can allocate extra mental resources to keep performance high for adequate responses to a critical traffic situation. While adaptation of driving behavior is immediately visible, adjustments of mental effort (as well as a driver’s mental state in general) are not. Also, increasing mental fatigue or attentional disengagement when driving on a monotonous road are not directly measurable, but they may have negative consequences for traffic safety. Therefore, monitoring a driver’s mental state during driving can help us to learn more about age-related differences in traffic-related performance and hidden processes of compensatory activity (for review Da Silva, 2014).

A powerful approach to objectively determine mental states while driving are physiological measures (De Waard, 2002; Brookhuis and de Waard, 2010; Borghini et al., 2014). Especially neurophysiological measures like the brain oscillatory activity derived from the EEG have a long tradition. Here, the overall oscillatory power as well as the relative power in specific frequency bands like the Theta band (4–7 Hz) and Alpha band (8–12 Hz) are of special interest, as these are assumed to reflect different mental states. *Decreased* Alpha power is usually regarded as a marker of allocation of attention (Herrmann and Knight, 2001) and higher working memory demands (Klimesch, 2012). In contrast, *increased* Alpha power is related to mental fatigue, as well as to attentional withdrawal and disengagement (Hanslmayr et al., 2012; Wascher et al., 2014, 2016) as observed during monotonous and boring tasks (Borghini et al., 2014). Accordingly, increases in Alpha power during monotonous driving situations and low perceptual demands may reflect periods of inattention and mind-wandering (Lin et al., 2016). Activation in the Theta band, on the other hand, is assumed to reflect aspects of executive functioning and—more generally—cognitive control (Cavanagh and Frank, 2014; Cavanagh and Shackman, 2015). Accordingly, Theta power is usually increased with higher task demands (Jensen and Tesche, 2002; Onton et al., 2005) and—when time on task increases—with the effort to keep performance high (Wascher et al., 2014; Arnau et al., 2017).

In a recent driving simulator study, we employed oscillatory EEG measures to investigate underlying cognitive processes that may explain inter-individual variability in driving performance (Karthaus et al., 2018). In a lane-keeping scenario, in which younger and older drivers had to respond to variable levels of crosswind by compensatory steering movements, both age groups showed comparable overall performance. However, the analysis of Alpha and Theta power suggested subtle differences in driving styles, on the one hand within the older group and, on the other hand, between the younger and older drivers. In accordance with previous results (Garcia et al., 2017), these driving styles could be described as either re-active or pro-active: while re-active driving was characterized by high driving lane variability and higher Alpha power, pro-active driving was indicated by low driving lane variability and lower activity in Alpha (and Beta) band. The latter has been associated with a more alert mental state, a better anticipation and active use of ongoing sensory driving information, and a more proactive planning of future responses. The re-active driving style, in contrast, led to situations in which the driver rather re-acts to environmental information, resulting in delayed compensatory steering activity (see also Braver, 2012).

Whether a driver uses a more re-active or pro-active driving style does not only depend on the driver him/herself, but also on the external conditions, i.e., the degree to which a situation in principle can be controlled. A highly controllable situation enables the anticipation and planning of future actions (e.g., steering movements when driving on a curvy road under good visibility conditions), whereas a poorly controllable situation forces the driver to respond exclusively to unpredictable outer stimulation (Garcia et al., 2017). Depending on the driving situation, the drivers’ mental states may vary profoundly: a recent EEG study, in which pro-active and re-active driving scenarios were contrasted by employing either a curve-taking or crosswind-compensation task, it could be demonstrated that the latter task results much faster in a mental state of attentional disengagement and withdrawal of attentional resources (as indicated by an increasing Alpha). Taking bends, in contrast, was associated with a more focused driving activity, as indicated by a higher relative Theta power in general, and an additional increase in Theta power in narrow curves (Wascher et al., 2018). This higher Theta power can be interpreted as the consequences of the need for higher cognitive control in more demanding driving situations (see also Cavanagh and Frank, 2014). In line with this, higher steering demands in a pro-active driving scenario clearly resulted in increases in mental effort (Dijksterhuis et al., 2011).

Mental workload, task demands and driving performance are closely interrelated (Da Silva, 2014). For driving safety, a core question is therefore to what degree a driver actively uses the information provided by the environment and whether he or she adequately and flexibly adapts the processing of this information to a current driving situation. This critically depends on the interaction of: (a) the driving situation that might be more or less controllable; (b) the current workload of a given situation; and (c) the driver’s individual mental capacities that might be reduced due to, e.g., temporal states of fatigue or boredom, or long-term age-related declines in cognitive functioning. The present study investigated this interplay in a pro-active lane-keeping driving task, in which younger and older drivers had to keep track on a curvy road. The interaction of age, varying degrees of task workload (operationalized by bends of different radii), and variations in mental effort over time was tested by analysis of behavioral, neurophysiological and subjective measures. In particular, steering variability was analyzed as a measure of driving effort, with higher variability being associated with higher effort. By the analysis of oscillatory power in the Alpha and Theta bands, mental states of attentional withdrawal and effort were determined.

In addition to brain oscillatory power, event-related brain responses (ERPs) were analyzed that offer a further approach to objectively measure mental effort and task load of a primary task. Therefore, an auditory oddball paradigm has been applied in which ERPs were measured to a stream of task-irrelevant tone stimuli consisting of frequently presented standard and rare deviant stimuli. Two different fronto-central ERP components were analyzed, the mismatch negativity (MMN) reflecting the automatic context-dependent pre-attentive information processing of deviant tone stimuli (irrespective of the subject’s focus of attention; for review Näätänen et al., 2007), and the P3a indicating an involuntary shifting of attention towards a deviant stimulus (e.g., Näätänen, 1992). Results of previous studies showed that MMN and P3a provide suitable approaches to mental states, like mental fatigue (Massar et al., 2010; Yang et al., 2013) or attentional load (Yucel et al., 2005; Zhang et al., 2006) as well as to task workload and time on task (Kramer et al., 1995; Wascher et al., 2016). In particular, in a steering-task paradigm, the P3a elicited by task-irrelevant auditory probes was reduced with higher steering difficulty, suggesting the P3a to be an indirect measure for evaluating mental workload (Brouwer et al., 2012; Scheer et al., 2016). Thus, in the present study, higher amplitudes of MMN and P3a would indicate a deeper processing of the task-irrelevant probes, either at a pre-attentive or attentional level, potentially impairing performance in the driving task.

## Materials and Methods

### Participants

Sixteen younger (eight female, mean age 24.1 years, age range 20–30 years) and 16 older (eight female, mean age 63.3 years, age range 55–69 years) active car drivers (at least two drives per week during the last 3 years) participated in the study. The data of one (older) participants were excluded from analysis due to profound EEG artifacts. As could be expected, the older drivers hold their driving licenses longer than the younger ones (young: 6.7 years, SE 0.7 years; older: 43.7 years, SE 1.7 years; *t*_(29)_ = 20.66; *p* < 0.001), but the two groups did not differ in their mean annual mileage (young: 12207 km, SE 4435 km; older: 12455 km, SE 1609 km; *t*_(24)_ = 0.05; *p* > 0.05; reduced number of participants). All participants had normal or corrected-to-normal vision, and none of them reported any known neurological or psychiatric disorder. They received 30 € for participation in the experiment and provided written informed consent prior to entering the experiment. This study was carried out in accordance with the recommendations of Code of Ethics of the World Medical Association (Declaration of Helsinki). The protocol was approved by the local Ethical Committee of the Leibniz Research Centre for Working Environment and Human Factors, Dortmund, Germany. All subjects gave written informed consent in accordance with the Declaration of Helsinki.

### Task and Procedure

The experiment took place in a static driving simulator, consisting of three 32 inch displays and 200 degrees horizontal field of view (ST Sim; ST Software B.V. Groningen, Netherlands). The participants’ task was to keep a car on track on a one-lane road with curves of varying radii. The driving environment consisted of monotonous grassland without any additional visual distraction. The driving speed was held constant at 31 mph to prevent the participants from compensatory slowing down in narrow curves. The radii of the curves varied randomly every 2 min between three levels (task load: low, middle, high): At the low level, there was a straight road without curves. At the high level, the curves were adjusted to radii that allowed the participants to keep the car on track for about 95% of the time (as determined in a pilot study). At the middle level, the curve radii were distributed in between the low and high levels. Left and right turns varied randomly within each curve segment. To smooth the transfer between adjacent segments and to avoid abrupt changes, 1-s transfer-intervals were introduced. Three different curve segments were combined to triplets in randomized order, each lasting for 6 min. The first triplet was used to familiarize the participants with the task. This practice triplet was followed by nine experimental triplets that were separated into three blocks. All in all, the experimental blocks lasted for 54 min without any break or interruption.

During the entire experiment a random sequence of short auditory stimuli was presented via two broad-band loudspeakers in front of the participants at a sound level of 70 dB (A). The duration of each stimulus was 100 ms (including 5 ms rise and fall times) and the interstimulus-onset interval was 1,000 ms. The majority of the stimuli (80%) were standards intermixed with 10% higher and 10% lower deviants. The standard stimulus was a harmonic tone composed of three sinusoidal partials of 500, 1,000 and 1,500 Hz, with the intensity of the second and third partials being lower than that of the first partial by 3 and 6 dB, respectively. The two deviant stimuli differed from the standard stimulus in frequency, being either 10% higher (partials: 550, 1,100, 1,650 Hz) or lower (450, 900, 1,350 Hz) than the standard. The tones represented irrelevant distractor stimuli that should be ignored by the participants.

In order to measure possible changes in subjective fatigue over the driving session, the participants filled out the German version of the Stanford Sleepiness Scale (Hoddes et al., 1973) immediately before and after the driving session.

### Data Recording

EEG was recorded by 64 scalp electrodes placed according to the International 10-10 system (electrode impedance below 10 kΩ) and a “BioSemi active 2” amplifier (BioSemi, Netherlands; sampling rate 2,048 Hz, bandwidth DC—140 Hz). Six additional electrodes positioned around both eyes were used for electrooculography to measure horizontal and vertical eye positions. Two additional electrodes were placed on the left and right mastoids.

### Data Analysis

#### Behavioral Data

Two different behavioral measures were analyzed, the time off track and the steering variability. Time off track was defined as the percentage of time that the car left the track. It was used as main index for individual driving accuracy. Steering variability was defined as the steering activity in degrees per second and was operationalized as an index for workload (Verwey and Veltman, 1996). Both parameters were entered into mixed-design ANOVAs with the between-subject factor Age (2; young, old) and the within-subject factors Task Load (3; low, medium, high) and Time on Task (3; Blocks 1, 2, 3).

#### EEG Data

After re-referencing the EEG data to common average reference, a bandpass filter between 0.1 Hz and 35 Hz was applied. Broken channels were detected and excluded based on kurtosis and probability criteria. Afterwards, the filtered data were resampled to 128 Hz (dataset 1) and epoched into segments ranging from −600 ms to 1,200 ms with respect to the onset of the sound stimuli. For the ERP analysis, 1,024 Hz sampled data (dataset 2) were segmented, also ranging from −600 ms to 1,200 ms, and a baseline ranging from −200 ms to 0 ms was subtracted. Segments containing artifacts were identified in dataset 1 and removed from both datasets 1 and 2. An independent component analysis (ICA) was performed on dataset 1 and ICs reflecting artifacts were identified using ADJUST (Mognon et al., 2011). The IC weights were then copied to dataset 2 to again remove artifactual ICs from both datasets.

The spectral properties of the EEG were obtained by calculating Fast Fourier Transformations on dataset 1. Due to substantial differences in the raw spectra between the two age groups, a two-step analysis was chosen. First, to address the different levels in general power, total power between 3 Hz and 30 Hz was calculated. Thereafter, the mean power was extracted for the Theta band (4–7 Hz) and the Alpha band (8–12 Hz) to subsequently compute relative power values. The proportional contribution of Theta and Alpha power to total power was entered into analyses.

Due to relatively liberal criteria of the statistical rejection of segments, an additional amplitude criterion was applied to the data (maximum voltage difference of ±50 μV per segment). To compute the deviance-related MMN and P3a components, difference waveforms were calculated by subtracting the standard-tone ERPs from the deviant-tone ERPs. For analysis of MMN and P3a amplitudes, the fronto-central FCz electrode was chosen where the most prominent responses are usually obtained (for reviews see Escera et al., 2000; Näätänen et al., 2007). The MMN and P3a amplitudes were calculated as a mean voltage within the 40-ms period centered at the peak latencies in the grand-average waveforms (MMN: 125 ms; P3a: 230 ms; relative to tone onsets).

It should be noted that the sequence of the tone stimuli temporarily overlapped with the driving task, and that the oscillatory measures were computed in epochs, in which the cortical processing of the auditory standards and deviants took place. Alpha and Theta frequency bands represent important portions of auditory event-related oscillations (e.g., Kolev and Yordanova, 1997; Yordanova et al., 2000) and possible effects of these event-related oscillations might be assumed. However, given that the tone sequence was kept constant throughout the driving session, and because the analysis was mainly focused on within-subjects effect of task workload and time on task as well as the interaction of these factors with age, such effects of event-related oscillations should not play a significant role for the analysis of Alpha and Theta power measures.

Total power, percentages of total power in Theta and Alpha bands (relative power), and MMN and P3a amplitudes were entered into mixed-design ANOVAs with the between-subject factor Age (2; young, old) and within-subject factors Task Load (3; low, medium, high) and Time on Task (3; Blocks 1, 2, 3). The statistical analysis of the spectral power of the EEG based upon the averaged spectrograms of four anterior channels (F1, Fz, F2, FCz) and four posterior channels (PO3, POz, Pz, PO4), respectively. Average measures of electrode patches were used in order to gain a better stability of these measures by accounting for minor topographical deviations of spectral power across subjects. Anterior and posterior electrode patches were chosen as spectral power modulations at these locations have been linked to changes in mental states affecting performance. Theta power, especially at frontal recording sites, was shown to reflect cognitive effort and the exertion of cognitive control, thus also reflecting task demands (Jensen and Tesche, 2002; Maurer et al., 2014). Posterior alpha power, on the other hand, has been linked to cognitive disengagement and sensory withdrawal (Hanslmayr et al., 2011). In the context of time on task effects, recent studies report reliable increases of alpha power at anterior leads (Barwick et al., 2012; Wascher et al., 2014; Fan et al., 2015). False discovery rate (FDR) correction was applied to account for this multiple testing, and only FDR-corrected *p*-values are provided. Effect sizes were provided for interpretation of the practical significance of the results, using the partial eta-squared coefficient ($\eta_{\text{p}}^{2}$).

#### Subjective Data

Sleepiness ratings on the Stanford Sleepiness Scale were entered into mixed-design ANOVAs with the between-subject factor Age (2; young, old) and the within-subject factors Time (2; pre, post). In addition, changes in subjective mental states over the course of the driving session were computed as relative differences in sleepiness before and after driving, using the formula (Post − Pre/Pre * 100%).

## Results

### Behavioral Data

Time off track was slightly increased with higher Task Load (*F*_(2,58)_ = 2.75; *p* = 0.09; $\eta_{\text{p}}^{2}$ = 0.09) but did not depend on Time on Task or Age. None of the interactions reached significance (all *p* > 0.22; all $\eta_{\text{p}}^{2}$ < 0.05).

**Figure 1:**
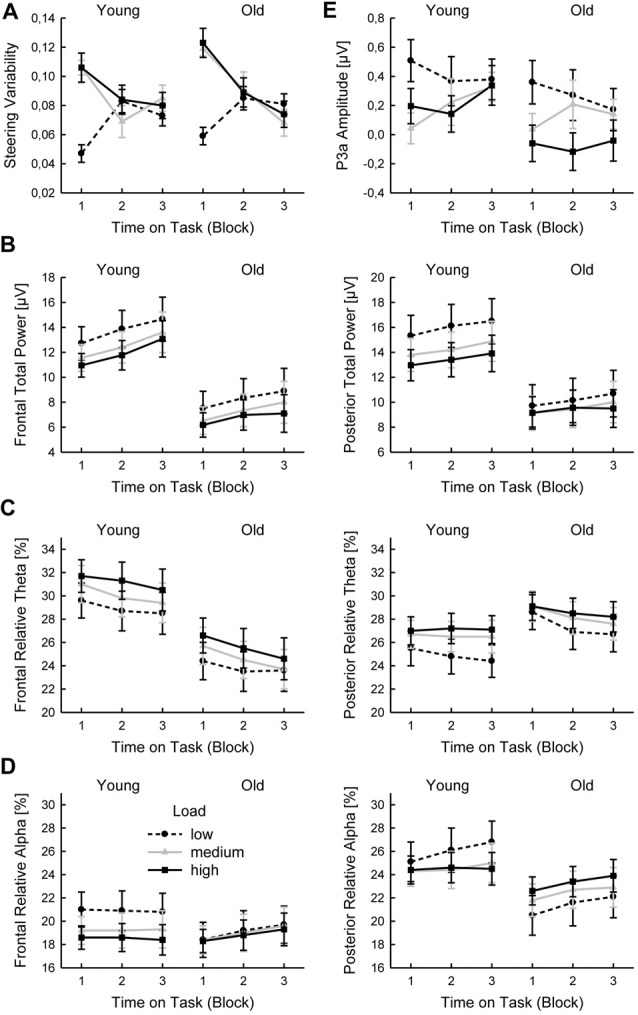
Behavioral and EEG parameters (mean values and standard errors of means): steering variability **(A)**, total oscillatory power **(B)**, relative Theta power **(C)**, relative Alpha power **(D)** and P3a amplitude **(E)** as function of Time on Task and Task Load, shown separately for young and old participants.

Steering variability was overall higher with higher Task Load (*F*_(2,58)_ = 9.08; *p* < 0.005; $\eta_{\text{p}}^{2}$ = 0.24) and decreased with Time on Task (*F*_(2,58)_ = 7.77; *p* < 0.005; $\eta_{\text{p}}^{2}$ = 0.21). Also, there was an interaction of Task Load and Time on Task (*F*_(4,116)_ = 12.72; *p* < 0.001; $\eta_{\text{p}}^{2}$ = 0.31) that was due a decrease of steering variability with Time on Task for medium and high task load, but rather an increase for low task load (Figure 1A). There was no main effect of Age (*F*_(1,29)_ = 1.47; *p* = 0.23; $\eta_{\text{p}}^{2}$ = 0.05) and no further interaction (all *p* > 0.10; all $\eta_{\text{p}}^{2}$ < 0.09).

### EEG Data

#### Total Power

The total power increased significantly with Time on Task (frontal: *F*_(2,58)_ = 11.66; *p* < 0.005; $\eta_{\text{p}}^{2}$ = 0.29; posterior: *F*_(2,58)_ = 8.00; *p* < 0.005; $\eta_{\text{p}}^{2}$ = 0.22) and decreased with increasing Task Load (frontal: *F*_(2,58)_ = 24.64; *p* < 0.001; $\eta_{\text{p}}^{2}$ = 0.46; posterior: *F*_(2,58)_ = 9.85; *p* < 0.005; $\eta_{\text{p}}^{2}$ = 0.25; Figure 1B). Total power was stronger in the younger than in the older group (frontal: *F*_(1,29)_ = 7.75; *p* < 0.05; $\eta_{\text{p}}^{2}$ = 0.21; posterior: *F*_(1,29)_ = 5.24; *p* < 0.05; $\eta_{\text{p}}^{2}$ = 0.15).

#### Relative Theta Power

The relative Theta power decreased with Time on Task (frontal: *F*_(2,58)_ = 12.58; *p* < 0.001; $\eta_{\text{p}}^{2}$ = 0.30; posterior: *F*_(2,58)_ = 8.28; *p* < 0.005; $\eta_{\text{p}}^{2}$ = 0.22) and was increased with increasing Task Load (frontal: *F*_(2,58)_ = 22.99; *p* < 0.001; $\eta_{\text{p}}^{2}$ = 0.44; posterior: *F*_(2,58)_ = 18, 10; *p* < 0.001; $\eta_{\text{p}}^{2}$ = 0.38). The decrease of posterior relative Theta power with Time on Task was more pronounced with lower than with higher Task Load (Time on Task by Task Load interaction: *F*_(4,116)_ = 4.02; *p* < 0.01; $\eta_{\text{p}}^{2}$ = 0.12; Figure 1C). Frontal relative Theta power was slightly stronger in the younger than in the older group (*F*_(1,29)_ = 5.58; *p* = 0.05; $\eta_{\text{p}}^{2}$ = 0.16).

#### Relative Alpha Power

The posterior relative Alpha power increased with Time on Task (*F*_(2,58)_ = 5.11; *p* < 0.05; $\eta_{\text{p}}^{2}$ = 0.15), and the frontal relative Alpha power was decreased with higher Task Load (*F*_(2,58)_ = 9.10; *p* < 0.01; $\eta_{\text{p}}^{2}$ = 0.24). The latter effect on frontal relative Alpha power was more pronounced in the younger than in the older group (Age by Task Load interaction: *F*_(2,58)_ = 5.79; *p* < 0.05; $\eta_{\text{p}}^{2}$ = 0.17; Figure 1D). Accordingly, separate ANOVAs for the two groups indicated a significant effect of Task Load for the younger (*F*_(2,60)_ = 18.41; *p* < 0.001; $\eta_{\text{p}}^{2}$ = 0.55), but not the older group (*F*_(2,56)_ = 0.18; *p* = 0.84; $\eta_{\text{p}}^{2}$ = 0.01), indicating that the increase of relative Alpha with lower Task Load was confined to the younger drivers. On posterior side, the effect of Task Load on relative Alpha power was also modulated by Age (Age by Task Load interaction: *F*_(2,58)_ = 5.85; *p* < 0.05; $\eta_{\text{p}}^{2}$ = 0.17). Here, separate ANOVAs indicated a significant interaction of Task Load and Time on Task for the younger group (*F*_(4,60)_ = 2.74; *p* < 0.05; $\eta_{\text{p}}^{2}$ = 0.16), demonstrating a more pronounced increase in relative Alpha power over time with lower than with higher Task Load. In contrast, the older group showed an overall decrease in relative Alpha power with lower Task Load (*F*_(2,56)_ = 4.46; *p* < 0.05; $\eta_{\text{p}}^{2}$ = 0.24).

#### MMN and P3a

The MMN amplitude (mean −0.33 μV, SE 0.05 μV) did not depend on Time on Task or Task Load and did not differ between age group (all *p* > 0.21; all $\eta_{\text{p}}^{2}$ < 0.06). The P3a amplitude decreased with higher Task Load (*F*_(2,58)_ = 6.33; *p* < 0.005; $\eta_{\text{p}}^{2}$ = 0.18; Figure 1E) and was slightly higher in the younger group (*F*_(1,29)_ = 3.48; *p* = 0.07; $\eta_{\text{p}}^{2}$ = 0.11).

### Relationship Between Steering Variability and EEG

In order to evaluate possible relationships between driving performance and mental states, steering variability and EEG measures were averaged across Task Load and Time on Task, and a correlation was calculated for total power, relative Theta and Alpha power, and ERPs separately. The Kendall’sTau correlation coefficient was used as a non-parametric test for the statistical dependance of behavior and EEG measures.

#### Total Power

There were no significant correlations of steering variability and total power, neither for the younger (frontal: *τ* = 0.217; posterior: *τ* = 0.265), nor for the older group (frontal: *τ* = −0.067; posterior: *τ* = −0.105; all *p* > 0.14).

#### Relative Theta Power

**Figure 2:**
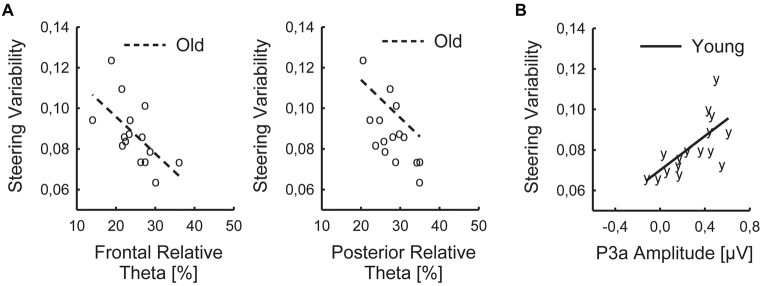
Significant relationships of behavioral and EEG parameters: Individual values of steering variability, frontal and posterior relative Theta **(A)**, and P3a amplitude **(B)**, averaged across Task Load and Time on Task, shown for young (y) and old (o) participants with regression lines.

A significant negative correlation of steering variability and relative Theta power occurred in the older group (frontal: *τ* = −0.524; *p* = 0.024; posterior: *τ* = −0.467; *p* = 0.030; FDR-corrected *p*-values), indicating that higher Theta power was associated with lower steering variability (Figure 2A). No such relationship was found in the younger group (frontal: *τ* = −0.150; posterior: *τ* = 0.100; both *p* > 0.55).

#### Relative Alpha Power

There was no correlation of steering variability and relative Alpha power, neither for the younger group (frontal: *τ* = −0.050; posterior: *τ* = 0.133), nor for the older group (frontal: *τ* = −0.143; posterior: *τ* = 0.048; all *p* > 0.45).

#### MMN and P3a

There was a significant positive correlation of steering variability and P3a amplitude in the younger group (*τ* = 0.550; *p* = 0.012), indicating that a more pronounced P3a was associated with higher steering variability (Figure 2B). In the older group, the correlation of steering variability and P3a slightly failed to reach significance (*τ* = 0. 333; *p* = 0.083). In addition, there was a trend to a correlation of steering variability and MMN (younger: *τ* = 0.383; *p* = 0.051; older: *τ* = 0.410; *p* = 0.051; FDR-corrected *p*-values).

### Subjective Data

**Figure 3:**
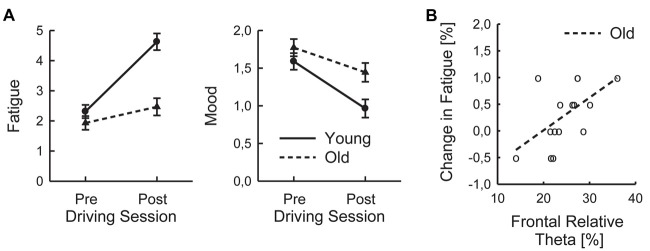
Subjective parameters: reported pre and post driving fatigue ratings **(A)** and individual changes in fatigue and frontal relative Theta **(B)** of old (o) participants (as averaged across Task Load and Time on Task) with regression lines.

The analysis of the pre and post ratings of fatigue indicated an increase in subjective sleepiness over the course of the driving session (*F*_(1,29)_ = 42.12; *p* < 0.001; $\eta_{\text{p}}^{2}$ = 0.59; Figure 3A). In addition, the younger group scored higher in overall sleepiness (*F*_(1,29)_ = 16.46; *p* < 0.001; $\eta_{\text{p}}^{2}$ = 0.36) than the older group. A significant interaction of Age and Time on Task on sleepiness (*F*_(1,29)_ = 16.46; *p* < 0.001; $\eta_{\text{p}}^{2}$ = 0.36) was due to a higher increase in sleepiness in the younger, than older, group.

In order to test whether relative frontal Theta power and P3a amplitude, which turned out to be related to steering variability in the older (respective younger) group, were also associated with the subjective fatigue, the relative changes in sleepiness over the driving session were computed and correlations were determined for frontal relative Theta power and P3a amplitude for both groups separately. In the older group, a significant correlation was found for frontal relative Theta (*τ* = 0. 490; *p* = 0.036; FDR-corrected p-value; Figure 3B), indicating that higher relative Theta power during the driving session was associated with a stronger increase in sleepiness. The relationship of frontal relative Theta and change in sleepiness in the younger group did not reach significance (*τ* = 0.188; *p* = 0.315). No significant correlations of P3a and subjective measures were found, neither for the younger (*τ* = 0.103), nor for the older group (*τ* = 0.000; both *p* > 0.58).

## Discussion

Goal of the present study was to explore the interaction of task workload, variations in mental effort, and age-related difference in performance in a simulated (lane-keeping) driving task, using electrophysiological EEG and ERP measures. Lane keeping in general was efficient in younger and older drivers. There was a slight increase in time off track in tighter bends that could be expected. However, there were no significant changes over time, neither in the younger, nor in the older group. Steering variability on curvy road sections was increased relative to straight sections in both groups but decreased with time on task. This could be due to learning effects resulting in a more and more experienced anticipation of steering behavior in dependance of the curve radius. In this regard, it should be noted that the driving scenario allowed for a pro-active driving strategy, in which the future steering behavior could be dynamically adapted to the course of the road in an anticipatory manner. This is in sharp contrast to the cross-wind scenario of our previous study (Karthaus et al., 2018), in which the driver could only react to the different degrees of crosswind but had no chance to prepare for an upcoming steering event over a longer time frame.

The analyses of the EEG oscillatory data indicated an overall stronger total power in the younger group. Such age-related differences in oscillatory activity have been reported in a number of previous studies (e.g., Polich, 1997; for review Klimesch, 1999), but the relationship between EEG measures, cognitive performance, and age are still poorly understood and obviously depend on the cognitive functions studied (e.g., Vlahou et al., 2014; Trammell et al., 2017). Beside this age effect, there was an increase in total power over time and a decrease with increasing workload. These effects of time on task and workload were found for frontal and posterior positions and appear plausible, given that strong oscillatory activity in lower bands (in particular the Alpha band) reflects mental states of boredom and attentional disengagement (e.g., Klimesch, 1999, 2012; Hanslmayr et al., 2012; Wascher et al., 2016) as usually observed during monotonous and boring tasks (Borghini et al., 2014). These mental states might be more pronounced on straight road sections and after the participants had become more familiar with the driving task because of automatization of a well-learned task.

A quite similar overall pattern was found for the relative Alpha power. However, there were differences between the two age groups: stronger frontal relative Alpha power and increases in posterior relative Alpha power on straight road sections, relative to curvy sections, were only observed in the younger group. In contrast, in the older group frontal relative Alpha power did not differ between task loads, and posterior relative Alpha power was even smaller on straight road sections. Thus, the afore-mentioned attentional disengagement was only found in the younger group, whereas the older drivers showed less signs of boredom and mental fatigue even when the task was less demanding. This observation can be interpreted with regard to the decline-compensation hypothesis (Cabeza et al., 2002), assuming that older adults maintain or even increase their mental effort to counteract possible age-related neurocognitive declines. Accordingly, neurophysiological studies have frequently demonstrated greater activation especially in frontal brain areas in older adults than in younger adults, even when the performance did not differ (for review Reuter-Lorenz and Cappell, 2008).

In contrast to relative Alpha power, relative Theta power decreased over time and was stronger with increasing workload. This observation is well in line with the view that Theta power reflects mental effort (e.g., Jensen and Tesche, 2002; Onton et al., 2005; Cavanagh and Frank, 2014; Cavanagh and Shackman, 2015; Arnau et al., 2017), which should decrease over time due to learning and automatisms, but which should increase on demanding road sections. While this interplay of time on task, workload, and Theta power did not differ between the two age groups, a significant negative relationship of relative Theta power and steering variability was found only in the older group: Older drivers with higher Theta power showed a lower steering variability. Assuming that steering variability represents a measure for workload (Verwey and Veltman, 1996), and frontal Theta power constitutes an indicator for mental effort, this negative correlation does not appear plausible at first sight. However, for the interpretation of the present relationship, it should be considered that in a pro-active driving task lower steering variability reflects a more adequate steering behavior that requires (and results from) a better anticipation of the ongoing and future road course (Garcia et al., 2017). In other words: to perform the task successfully, the driver had to perceive the direction and strength of the curves continuously, respond by steering movements, monitor the movements of the car relative to the driving lane, and correct the steering movement if necessary. Higher steering variability is therefore the consequence of deficiencies in this ongoing loop of perceiving, responding, and correction. Lower steering variability, on the other hand, should be associated with higher mental effort in the proactive driving task, as indicated by higher frontal Theta power.

Interestingly, the correlation of steering variability and relative Theta power was significant only in the older group. The fact that the relationship of steering performance and mental effort exclusively occurred in the older group is—on the one hand—in line with the decline-compensation hypothesis (Cabeza et al., 2002). On the other hand, the differences between the age groups might reflect the higher inter-individual variability in elderly that is usually found (e.g., Hultsch et al., 2002). Higher differences in steering variability in the older group might promote correlations with brain measures on a between-subject level. In addition to differences *between* the older drivers (rather suggesting individual longer-term driving strategies), the question of whether a similar relationship exists on a within-subject level would also be of interest, as this would reflect short-term variability in the interplay of task workload, mental effort and driving outcome. Here, it would be expected that current states of higher mental effort should be accompanied by lower actual steering variability. In order to address this question, recent research approaches using ongoing EEG recordings have been proposed for the assessment of mental workload, fatigue and drowsiness in car drivers (e.g., Borghini et al., 2014; Charbonnier et al., 2016; Kong et al., 2017).

The assumption that better steering behavior of older drivers was associated with higher mental effort is further supported by the significant correlation of relative Theta power and the increase in the subjective measure of fatigue: Older drivers showing higher relative Theta power (indicating higher mental effort while driving) reported a higher pre-/post-increase on the sleepiness scale. Such an association of mental effort and relative Theta power has also been reported in previous studies (Klimesch, 1999; Smith et al., 2001; Smit et al., 2004). Although a similar relationship could be observed in the younger group (see Figure 3B), the correlation failed to reach significance. The younger group also reported an overall higher level of sleepiness that even increased more than that of the older group. This suggests a higher tendency of the younger group to respond to monotonous task with boredom and attentional withdrawal than the older group, as it was also found in a previous study (Arnau et al., 2017). This also corresponds nicely to the observation that an increase in posterior relative Alpha power on straight, less demanding road sections was only observed in the younger group (see above).

The analysis of the ERPs to deviance in the acoustic stimulation indicated an MMN and a P3a to the rare deviant tones, relative to the regular standard tone. The MMN is regarded as a correlate of deviance detection at an early, rather pre-attentive level, while the P3a reflects the allocation of attentional resources toward a change in the individual’s environment (Näätänen, 1992; for review Näätänen et al., 2007). In classical distraction paradigms, in which participants are instructed to attend to a task-relevant stimulus feature while ignoring task-irrelevant features, the P3a is usually assumed to reflect an involuntary shift of attention toward the distractor, away from the task at hand (Escera et al., 2000). In the present driving task, MMN and P3a can be regarded as correlates of detection of and attention to the deviant tone stimulus. The first process did not depend on workload and time on task (which could be expected, given that the MMN in passive auditory oddball paradigms can be found even in coma patients, e.g., Morlet and Fischer, 2014). The second one, however, decreased in amplitude with higher workload. This can be interpreted in the framework of a resource allocation approach (for review see Wickens, 2008), in which less allocation of cognitive resources to a secondary task shows that more resources are required by a primary task. In line with this hypothesis, diminished P3 amplitudes to task-irrelevant sounds have been observed in a primary steering task when steering demands increased (Scheer et al., 2016). In the present task setting, keeping track in narrow curves might tie up attentional resources, which are no longer available for attending to the changes in the tone stimuli. In this regard, it should be noted that MMN and P3a are determined as the difference waveforms of ERPs to deviant and standard tones. Thus, the workload effect on P3a amplitude does not simply mirror the pattern of total oscillatory power but reflects genuine workload-related differences in stimulus deviance processing. The slightly higher P3a amplitude in younger participants is in line with the literature (Polich, 1997). More importantly, the younger group showed a significant correlation of P3a amplitude and steering variability, suggesting that worse steering performance of younger drivers came along with a higher involuntary shift of attention toward the deviant tone stimuli. Conversely, younger drivers who attended less to changes in the environment showed a better driving performance. This relationship clearly stresses the implication of the P3a to task-irrelevant sound as an indirect measure for evaluating mental workload, which has also been shown previously (Scheer et al., 2016). Furthermore, assuming that shifts of attention toward task-irrelevant stimulation are closely related to distraction, the present results suggest that the susceptibility to distraction of younger and older drivers is based on different cognitive mechanisms.

As a final methodological remark, it should be noted that the most prominent results of the present study were found in relative values of Theta and Alpha power, whereas the analysis of total oscillatory power indicated only basic effects of workload and time on task. Thus, relative power in EEG bands appears to be the more fine-grained measure that should be considered in future studies.

## Conclusion

In the present pro-active driving scenario, younger and older drivers did not differ in behavioral measures of performance, i.e., in time off track and steering variability. However, electrophysiological measures demonstrated age-related differences in the way the two groups reached this goal: high performance was associated with increased mental effort (and a higher increase in fatigue) in the older group. Yet, high performance in the younger group was related to higher attentional focusing on the driving task (respective less attention to distractors). What do these results mean for traffic safety? On the one hand, it appears that older drivers invest extra mental resources to manage demanding (driving) tasks. Thus, they should always start a longer trip sufficiently rested and otherwise fit for driving. Also, it is advisable for them to take breaks regularly. Driver assistance systems detecting drowsiness can additionally help to avoid driving situations in which reduced mental resources might lead to critical driving behavior. On the other hand, younger drivers showing a tendency to attend to traffic-irrelevant stimulation should avoid being distracted by this information, especially in monotonous driving situations. Here, more initiatives towards younger drivers addressing the dangers of using mobile phones and communication devices while driving would be recommended.

## Author Contributions

SG, MK and EW: study conception and design. MK: acquisition of data. SG, MK, SA, JER and EW: analysis and interpretation of data; critical revision. SG and EW: drafting of manuscript.

## Conflict of Interest Statement

The authors declare that the research was conducted in the absence of any commercial or financial relationships that could be construed as a potential conflict of interest.
